# Perception of junior doctors about local induction in Birmingham and Solihull Mental Health Foundation Trust - A quality improvement project

**DOI:** 10.1192/bjo.2021.532

**Published:** 2021-06-18

**Authors:** Saima Jehanzeb, Kozara Nader, Ruth Scally

**Affiliations:** 1 Birmingham and Solihull Mental Health Foundation Trust; 2 Birmingham and Solihull Mental Health NHS Foundation Trust

## Abstract

**Aims:**

A quality improvement project was undertaken to understand the perception of trainees about the quality of the local induction delivered by Birmingham and Solihull Mental Health Foundation Trust (BSMHFT). The primary aim was to evaluate the current local induction programme, following concerns raised by previous trainees in National Training Survey (General Medical Council) and local inspection. Our secondary aim was to devise a revised induction programme based on the trainees’ identified needs.

**Method:**

Two anonymised questionnaire surveys were emailed to all Foundation Year Trainees, Core Psychiatry Trainees and General Practice Speciality Trainees working in BSMHFT, in December 2019 and March 2020, using trust survey monkey.

**Result:**

The overall response to survey was 60 percent. 44.44 percent of the responses came from Core Psychiatry Trainees, with 27.78 percent responses each from Foundation Year Trainees and GP Speciality Trainees. Local induction was defined as induction specific to place of work (47.06%), trust based induction (41.18%) or all of the above options (11.76%) by trainees. 83.33% of all trainees had received local induction, whereas 16.67% did not have any local induction at the start of their post. 11.12% trainees were very satisfied and 44.44% were satisfied with local induction. 72.22 percent of the trainees were informed about of the local induction, prior to starting the post.

33.3% trainees had a paper version, 22.22% had an electronic version of local induction pack, whereas 44.44% had no induction pack. 55.55% of those trainees who had an induction pack, 43.75% found it very helpful and 56.25% did not find it helpful.88.89% thought having a local induction would be helpful, whereas 11.11 percent did not feel it would help. 94.44% of the trainees completed a local orientation checklist with their consultants. Some of the trainees experienced difficulty in gaining access to electronic prescribing, electronic patient record system (RIO), and identity badges (ID) at the beginning of their post.

**Conclusion:**

11.12% trainees were very satisfied, 44.44% were satisfied, 22.22 % were neither satisfied nor dissatisfied and 22 % were dissatisfied, with local induction. 88.89% of the trainees thought having a local induction pack would be helpful. Based on the trainees identified needs we developed a template for local induction pack for each post. Clinical supervisors have agreed to take the lead in preparing the local induction pack specific to their post with trainees.We aim to repeat the survey after implementing the changes identified by trainees based on their training needs.

